# A Randomized Controlled Trial Comparing Collagen Matrix to Hemostatic Gelatin Sponge as Socket Seal in Alveolar Ridge Preservation

**DOI:** 10.3390/jcm13082293

**Published:** 2024-04-16

**Authors:** Célien Eeckhout, Lorenz Seyssens, Maarten Glibert, Laurens Keppens, Baptist Nollet, Martijn Lambert, Jan Cosyn

**Affiliations:** 1 Department of Periodontology and Oral Implantology, Oral Health Sciences, Faculty of Medicine and Health Sciences, Ghent University, C. Heymanslaan 10, B-9000 Ghent, Belgium; lorenz.seyssens@ugent.be (L.S.); maarten.glibert@ugent.be (M.G.); laurens.keppens@ugent.be (L.K.); baptist.nollet@ugent.be (B.N.); jan.cosyn@ugent.be (J.C.); 2 Department of Equal Lifelong Oral Health for All (ELOHA), Oral Health Sciences, Faculty of Medicine and Health Sciences, Ghent University, C. Heymanslaan 10, B-9000 Ghent, Belgium; martijn.lambert@ugent.be

**Keywords:** dental implant, alveolar ridge preservation, socket seal, collagen matrix, gelatin sponge

## Abstract

(1) **Objectives:** This study aimed to compare a collagen matrix to a hemostatic gelatin sponge as a socket seal in alveolar ridge preservation (ARP). (2) **Methods:** Systemically healthy patients planned for ARP at two sites with more than 50% of the buccal bone wall remaining after tooth extraction were eligible for inclusion. ARP involved socket grafting using collagen-enriched deproteinized bovine bone mineral. Sites were then randomly assigned to the test group (collagen matrix) or the control group (hemostatic gelatin sponge). The primary outcome was soft tissue thickness in the center of the site at 4 months, analyzed on cone-beam computed tomography. Secondary outcomes included the buccal and lingual soft tissue heights, horizontal bone loss, buccal soft tissue profile changes, wound dimensions, and Socket Wound Healing Score (SWHS). (3) **Results:** In total, 18 patients (12 females, 6 males) with a mean age of 57.3 years (SD 11.1) were included. Four months after ARP, the soft tissue thickness in the center of the site amounted to 2.48 mm (SD 0.70) in the test group and 1.81 mm (SD 0.69) in the control group. The difference of 0.67 mm (95% CI: 0.20–1.14) in favor of the collagen matrix was statistically significant (*p* < 0.009). The buccal soft tissue height was also statistically significantly higher for the collagen matrix (0.72 mm; 95% CI: 0.06–1.38; *p* = 0.034). A trend favoring the collagen matrix was found for the lingual soft tissue height (*p* = 0.066). No significant differences between the groups in terms of horizontal bone loss, buccal soft tissue profile changes, wound dimensions, and the SWHS were found. (4) **Conclusions:** The absence of significant differences in hard tissue outcomes suggests that both the collagen matrix and hemostatic gelatin sponge effectively sealed the extraction socket and supported bone preservation. However, the collagen matrix better maintained soft tissue dimensions. The clinical relevance of this finding with respect to the necessity for adjunctive soft tissue augmentation at the time of implant placement is yet to be studied.

## 1. Introduction

Alveolar ridge preservation (ARP) involves the insertion of bone graft material into the socket of an extracted tooth. Recent systematic reviews have demonstrated that ARP has the potential to reduce bone resorption by approximately 50% compared to what is usually observed after unassisted healing [1,2]. This outcome suggests that ARP is effective but not able to completely prevent postextraction bone remodeling. Socket sealing is an important aspect of ARP since it protects the underlying bone from bacterial contamination during the early stages of healing. A recent network meta-analysis identified coronal flap advancement as the socket seal technique with the highest probability of achieving ARP, closely followed by open healing with a barrier [3]. Since coronal flap advancement may readily result in complications with wound healing and unfavorable esthetics due to insufficient soft tissues at the time of tooth extraction [4], open healing with a barrier is preferred. Initially, a free gingival punch graft from the palate was used for this purpose [5]. However, a second surgical site is needed for graft harvesting, increasing postoperative morbidity [6,7], and scarring is often observed [8]. Therefore, biomaterials are widely used as socket seals nowadays, basically including hemostatic gelatin sponge, collagen matrix, collagen sponge, membranes, and acellular dermal matrix.

A low-cost socket seal for ARP is a hemostatic gelatin sponge (Spongostan Dental^®^ 1 × 1 × 1 cm, Ethicon, Johnson & Johnson, New Brunswick, NJ, USA). This porcine-derived biomaterial stimulates the aggregation of platelets and enhances fibrin linkage, which may contribute to the formation, stabilization, and maturation of the initial blood clot. Therefore, it is often applied in the extraction sockets of patients under anticoagulant therapy [9]. When used as a socket seal, the cube-shaped sponge is compressed to about 2 mm in thickness and trimmed to the required round shape with scissors. A hemostatic gelatin sponge has been recently compared to a free gingival punch graft as a socket seal in an RCT on ARP, yielding better bone preservation and less postoperative pain when using the hemostatic gelatin sponge [7].

A collagen matrix is also commonly applied as a socket seal in ARP. A porcine-derived non-cross-linked extracellular collagen matrix consisting of type I and III collagen was specifically introduced for socket sealing (Mucograft Seal^®^, Geistlich Pharma AG, Wolhusen, Switzerland). The structural characteristics of this matrix were designed to favor immediate blood clot stabilization and soft tissue cell ingrowth. The matrix has a diameter of 8 mm or 12 mm, which avoids trimming in most cases. With a thickness of 3 to 4 mm, it can be easily sutured at the soft tissue level. The collagen matrix has been compared to a free gingival punch graft as a socket seal in multiple RCTs on ARP, and the results have indicated similar bone preservation [6,10,11].

Even though the impact of different socket seals on bone preservation has been relatively well described, their influence on soft tissue dimensions has seldom been studied. However, the thickness of the soft tissue overlaying the extraction site is significant, as it influences the subsequent thickness of the buccal mucosa after implant placement, which is caused by buccal soft tissue displacement. As shown in systematic reviews, thicker soft tissues have been associated with superior implant esthetics [12] and healthier peri-implant conditions [13].

To the best of our knowledge, the collagen matrix has not been compared to the hemostatic gelatin sponge as a socket seal in ARP. The research hypothesis of this RCT was that the collagen matrix would result in thicker soft tissues than the hemostatic gelatin sponge due to its ideal thickness, structural characteristics, and presence of collagen in the grafting material.

## 2. Materials and Methods

### 2.1. Patient Selection

Patients planned for tooth extraction and ARP at two sites between July 2022 and December 2022 were screened by two clinicians (J.C. and M.G.) to participate in an intra-subject superiority RCT. Patients were selected based on inclusion and exclusion criteria.

The inclusion criteria were as follows:

- Aged 21 years or older;
- Good oral hygiene determined as a full-mouth plaque score ≤ 25% [14];
- Requirement for ARP after tooth extraction at two sites in the maxilla or mandible with more than 50% buccal bone remaining postextraction;
- Signed informed consent.

The exclusion criteria were as follows:

- Any systemic disease;
- Pregnancy;
- Smoking;
- Untreated periodontitis;
- Untreated caries lesions;
- Pus formation and/or ongoing infection surrounding the failing tooth;
- Midfacial recession at the failing tooth.

This study was approved by the Ethical Committee of the University Hospital in Ghent (BC-11345) and registered in ClinicalTrials.gov (NCT05423535) in June 2022. It was conducted in accordance with the ethical standards of the Declaration of Helsinki in 1975, as revised in 2013. The article was prepared according to the CONSORT statement for quality reporting of RCTs [15].

### 2.2. Randomization, Allocation Concealment, and Blinding

Since this was an intra-subject RCT, every patient received the test treatment as well as the control treatment. A coin flip decided which site in each patient was treated with the collagen matrix (test site) and which one with the gelatin sponge (control site). Group assignment was determined immediately after ARP and remained disguised for the evaluating examiners and statisticians, ensuring unbiased registrations and analyses, respectively.

### 2.3. Treatment Procedures and Postoperative Care

Patients were asked to take systemic antibiotics (amoxicillin 2 g) and anti-inflammatory medication (ibuprofen 600 mg) one hour before the procedure. Before the treatment, patients rinsed with a 0.12% chlorhexidine solution (Perio-aid^®^ Intensive Care, Dent-Aid Benelux, Houten, The Netherlands), and local anesthesia (Septanest special^®^, noradrenaline 1/100,000; Septodont, Saint Maur des Fossés, France) was administered.

No flap was made. When deemed necessary by the clinician, papillary incisions were made for slight reflection to facilitate the use of elevators without causing damage to the soft tissues. Buccal soft tissues were never raised. After thorough wound debridement and irrigation, the extent of missing bone was measured using a periodontal probe, rounded to the nearest 0.5 mm. Missing buccal bone was determined by measuring the vertical distance between the buccal bone crest and the midfacial soft tissue margin, subtracting 3 mm. Then, both alveolar sockets were filled with collagen-enriched deproteinized bovine bone mineral (C-DBBM, Bio-Oss Collagen^®^ 100 mg or 250 mg, Geistlich Pharma AG, Wolhusen, Switzerland) up to the lingual bone crest level. Thereupon, a coin was flipped to randomly assign either one of the two following treatment modalities:

- Test site: collagen matrix (Mucograft Seal^®^, Geistlich Pharma AG, Wolhusen, Switzerland);
- Control site: hemostatic gelatin sponge (Spongostan Dental^®^ 1 × 1 × 1 cm, Ethicon, Johnson & Johnson, New Brunswick, NJ, USA).

At both sites, the socket seal material was sutured with at least four single sutures (Seralon 6/0, Serag Weissner, Naila, Germany) to protect the underlying C-DBBM. Care was taken to ensure perfect approximation between the wound margins and the socket seal material. Both non-intact and intact alveoli were managed similarly. When a temporary removable denture was foreseen, it was trimmed to avoid pressure on the socket. 

**Figure 1:**
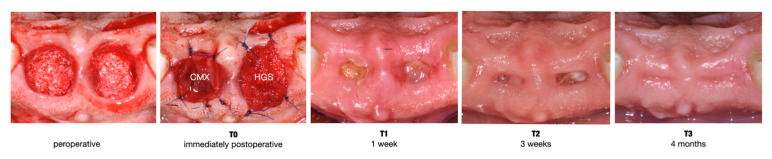
Clinical case showing an intraoperative image where both alveolar sockets were filled with collagen-enriched deproteinized bovine bone mineral (C-DBBM) up to the level of the lingual bone crest. To protect the underlying C-DBBM, a collagen matrix was sutured on top at the test site (left socket) and a hemostatic gelatin sponge at the control site (right socket) (T0). Wound healing is illustrated after 1 week (T1), 3 weeks (T2), and 4 months (T3).

Systemic antibiotics (amoxicillin 2 g) were continued for 4 days, and anti-inflammatory medication (ibuprofen 600 mg) was taken by the patient as deemed necessary. Patients rinsed with a 0.12% chlorhexidine solution twice daily for one week. Then, the sutures were removed. Figure 1 illustrates a clinical case.

### 2.4. Primary Outcome: Soft Tissue Thickness in the Center of the Site

**Figure 2:**
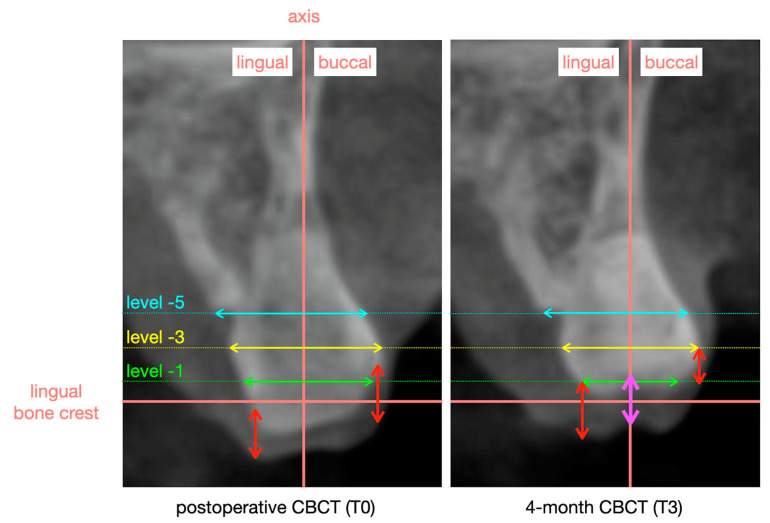
On the 4-month cone-beam computed tomography (CBCT) (T3) at the center of the extracted tooth, the following reference lines were established: the long axis of the extracted tooth, the lingual bone crest level perpendicular to the extracted tooth’s long axis, level −1 mm (green), level −3 mm (yellow) and level −5 mm (cyan) apical to the lingual bone crest and perpendicular to the extracted tooth’s long axis. Soft tissue thickness in the center of the site was measured along the vertical reference line of the extracted tooth’ long axis as the vertical distance between the alveolar process and the soft tissue outline (purple arrow). The bone width was registered at the different levels (1 mm level: green arrow, 3 mm level: yellow arrow, and 5 mm level: cyan arrow). The buccal and lingual soft tissue heights were measured as the vertical distance between the alveolar process and the soft tissue outline parallel to the vertical reference line of the extracted tooth’s long axis at the most buccal and lingual part of the alveolar process (red arrows). After all measurements were completed on the 4-month CBCT, the software switched to the superimposed postoperative CBCT (T0) image, maintaining all reference lines so that the same measurements could be performed.

Soft tissue thickness in the center of the site was evaluated on the 4-month CBCT (T3) in specialized software (Invivo6, Osteoid Inc., Santa Clara, CA, USA). CBCT images were captured with a ProMax 3D Max device (Planmeca, Helsinki, Finland) using standardized settings (90 kV, 6.3 mA, 9 s, voxel size 200 μm) and a consistent field of view (50 × 80 mm) for each patient. Lip retractors were used to enhance the visualization of the external soft tissue profile, and patients were instructed to pull their tongue back for the same reason. In the software, a reference line was drawn along the long axis of the alveolar socket. The soft tissue thickness in the center of the site was measured on this reference line as the vertical distance between the alveolar process and the soft tissue outline. Figure 2 illustrates the measurement protocol. Group allocation remained disguised for the evaluating examiner (L.K.) and statistician (C.E.), ensuring unbiased registrations and analyses, respectively.

### 2.5. Secondary Outcomes

*Changes in bone and soft tissue dimensions* were assessed on CBCT images taken at T0 (immediately postop) and T3 (4 months). Using specialized software (Invivo6, Osteoid Inc., Santa Clara, CA, USA), both CBCT images were superimposed, and reference lines were constructed, as described in Figure 2. The bone width was registered at three levels (1, 3, and 5 mm) from the lingual bone crest. The buccal and lingual soft tissue heights were measured as the vertical distance between the alveolar process and the soft tissue outline parallel to the vertical reference line at the most buccal and lingual part of the alveolar process. After all measurements were completed on the 4-month CBCT, the software switched to the superimposed postoperative CBCT (T0) image, maintaining all reference lines so that the same measurements could be performed. Finally, changes were calculated by subtracting the measurements at T3 from those at T0. Group allocation remained disguised for the evaluating examiner (B.N.) and statistician (C.E.), ensuring unbiased registrations and analyses, respectively.

*Changes in buccal soft tissue profile*: An intra-oral scan (Trios, 3 shape, Copenhagen, Denmark) was obtained at T0 (immediately postop) and T3 (4 months). The digital surface models were obtained in the STL (Surface Tessellation Language) format and imported into designated software (SMOP, version number 2.21.2, Swissmeda AG, Zurich, Switzerland) for profilometric analysis. A study-relevant area of interest (AOI) was selected on the buccal aspect of each site, extending from 0.5 mm below the soft tissue margin to 4 mm more apical. In the mesiodistal dimension, the AOI spanned from the mesial to the distal line angle. While the AOI varied between sites due to individual anatomical differences, it remained consistent within each site across time points. To assess changes over time, the digital surface models were superimposed using the best-fit algorithm, aligning them with unchanged adjacent tooth surfaces. The software calculated the mean volumetric change (mm^3^) within the AOI for each site from T0 to T3, which was then divided by the AOI to determine the mean change in the buccal soft tissue profile. Additional methodological details can be found in an earlier publication [16]. Group allocation remained disguised for the evaluating examiner (L.K.) and statistician (C.E.), ensuring unbiased registrations and analyses, respectively.

*Buccolingual and mesiodistal wound dimensions* were registered by the treating surgeon with a periodontal probe to the nearest 0.5 mm at T0 (immediately postop), T1 (1 week), and T2 (3 weeks).

The *Socket Wound Healing Score (SWHS) [17]* was determined at T1 (1 week), T2 (3 weeks) and T3 (4 months). The SWHS evaluates various parameters, including wound dehiscence, epithelialization, the quality of granulation tissue filling the postextraction socket, and the depth between early granulation tissue and the wound margin. Blinded examiners assessed the SWHS based on occlusal clinical images, assigning scores as follows: 0 = wound covered with keratinized gingiva, pink tissue color, no bleeding, continuous with healthy tissue; 1 = socket filled with organized granulation tissue, no bleeding, collapsed to a depth of 0–2 mm from the buccal gingival margin; 2 = socket filled with organized granulation tissue, ¼ to ½ of the wound showing red tissue color, no bleeding, collapsed to a depth of 2–4 mm from the buccal gingival margin; 3 = socket filled with unorganized granulation tissue, more than ½ of the wound showing red tissue color, bleeding, no evidence of acute infection; 4 = socket filled with foreign material (food, etc.) and showing signs of alveolitis.

### 2.6. Sample Size Calculation

A sample size calculation was conducted in SPSS Statistics 28 (IBM, New York, NY, USA) using the paired-sample *t*-test. The calculation aimed to detect a mean difference of 0.5 mm in the final soft tissue thickness between the test and control sites with a standard deviation of 0.6 mm [16]. Setting alpha at 0.05 and power at 0.8, the calculation suggested that 14 patients should be included. To account for potential dropouts, the sample size was increased to 18 patients.

### 2.7. Statistical Analysis

SPSS Statistics 28 (IBM, New York, NY, USA) was used for data analysis. Mean values, standard deviations, and 95% confidence intervals (CIs) were calculated per treatment group. The paired-sample *t*-test was used to compare the test and control sites at 4 months in terms of the soft tissue thickness in the center of the site and the buccal soft tissue profile. 

A linear mixed model was performed to analyze the other secondary outcomes (horizontal buccal bone loss at the different levels, the buccal and lingual soft tissue heights, wound dimensions, and the SWHS). The treatment group, time, and their interaction were modeled as fixed factors. Patient and tooth position were included as random factors. The estimated marginal means and 95% CIs were calculated per treatment group and per time point. The level of significance was set at 0.05.

## 3. Results

### 3.1. Patients

**Figure 3:**
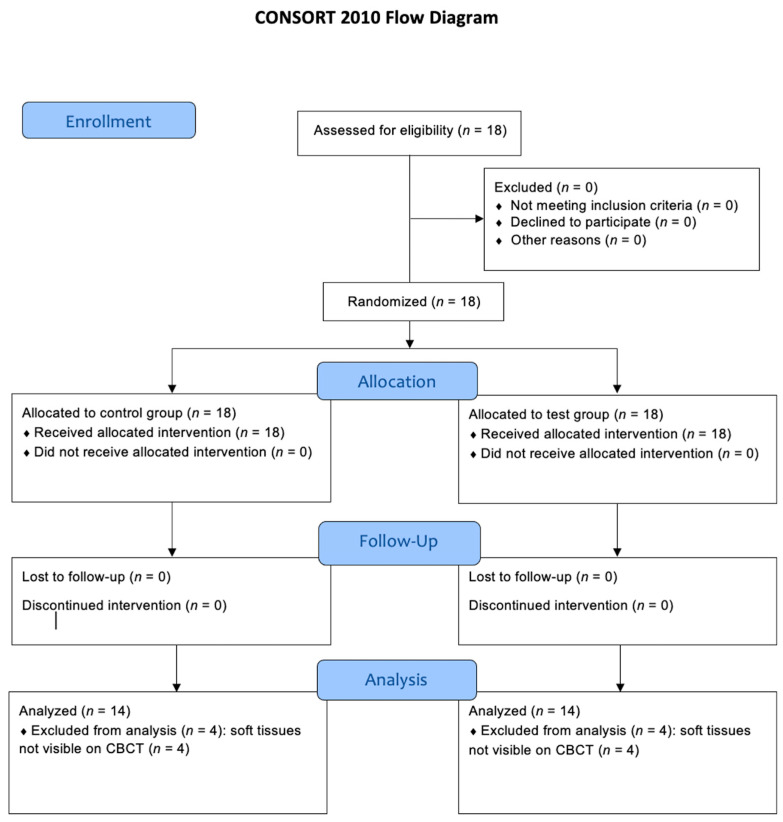
CONSORT flow diagram.

Figure 3 shows the CONSORT flow diagram. In total, 18 patients (12 females, 6 males) with a mean age of 57.3 years (SD 11.1) were assessed for eligibility. The number of treated patients was 18 (total), 17 (J.C.), and 1 (M.G.). 

The test treatment was performed at the following sites: six central incisor positions, one lateral incisor position, six canine positions, four premolar positions, and one molar position. The control treatment was performed at the following sites: four central incisor positions, two lateral incisor positions, seven canine positions, and five premolar positions. 

Buccal bone dehiscence was found after tooth extraction in two patients. In one patient, the buccal bone loss was 2.0 mm at both the test site and the control site. In another patient, the buccal bone loss amounted to 3.5 mm at the test site and 2.5 mm at the control site.

All patients could be re-examined at study termination. Yet, for the primary outcome, four cases had to be excluded from the analysis since the soft tissues were not clearly visible on CBCT.

### 3.2. Primary Outcome: Soft Tissue Thickness in the Center of the Site

The soft tissue thickness in the center of the site amounted to 2.48 mm (SD 0.70) in the test group and 1.81 mm (SD 0.69) in the control group. A statistically significant difference of 0.67 mm (95% CI: 0.20–1.14) between the groups was found (*p* < 0.009).

### 3.3. Secondary Outcomes

#### 3.3.1. Changes in Bone and Soft Tissue Dimensions

Table 1 shows the results of the horizontal changes in bone dimensions at level −1 mm, −3 mm and −5 mm apical to the lingual bone crest. Regardless of the level and group, statistically significant horizontal bone loss was observed. At level −1 mm, the most horizontal resorption was noted, measuring 2.67 mm in the test group and 2.70 mm in the control group. The difference of 0.03 mm (95% CI: −1.23–1.16) between the groups was not statistically significant (*p* = 0.958). At level −3 mm, the horizontal bone loss was 1.76 mm in the test group and 1.88 mm in the control group. The difference of 0.12 mm (95% CI: −0.90–1.13) between the groups was not statistically significant (*p* = 0.816). At level −5 mm, the horizontal bone loss was 1.12 mm in the test group and 1.32 mm in the control group. The difference of 0.20 mm (95% CI: −0.75–1.14) between the groups was not statistically significant (*p* = 0.679).

Table 1 shows the results on the buccal and lingual soft tissue heights. In the test group, the buccal soft tissue height increased by 0.07 mm, but this was not statistically significant (*p* = 0.772). In the control group, the buccal soft tissue height significantly decreased by 0.65 mm (*p* = 0.007). The difference of 0.72 mm (95% CI: 0.06–1.38) between the groups was statistically significant (*p* = 0.034).

The lingual soft tissue height increased by 0.32 mm in the test group (*p* = 0.240) and decreased by 0.38 mm in the control group (*p* = 0.160), yet both were not statistically significant. The difference of 0.70 mm (95% CI: −0.04–1.44) between the groups was not statistically significant (*p* = 0.066), yet there was a trend in favor of the test group.

#### 3.3.2. Changes in Buccal Soft Tissue Profile

A shrinkage in the buccal soft tissue profile was found in both groups at four months and amounted to 1.37 mm (SD 1.54) in the test group and 1.23 mm (SD 0.92) in the control group. The difference of 0.14 mm (95% CI: −0.64–0.91) between the groups was not statistically significant (*p* = 0.710). 

#### 3.3.3. Buccolingual and Mesiodistal Wound Dimensions

**Figure 4:**
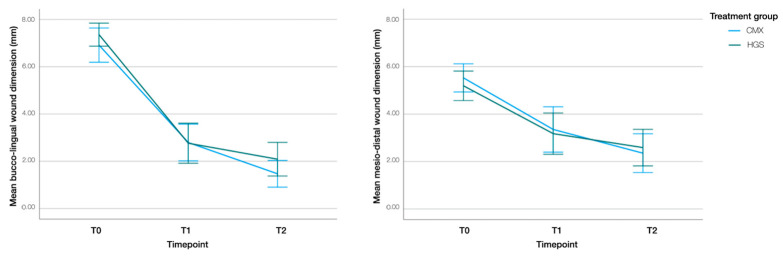
Buccolingual and mesiodistal wound dimensions at T0 (immediately postop), T1 (1 week), and T2 (3 weeks). Estimated marginal means and 95% confidence intervals are shown.

In both groups, the most significant reduction in wound dimensions occurred within the first week of healing (T0–T1) (Figure 4). In the test group, buccolingual and mesiodistal wound dimensions decreased by 4.09 mm (95% CI: 3.24–4.94; *p* < 0.001) and 2.13 mm (95% CI: 1.29–2.97; *p* < 0.001) between T0 and T1, respectively. Between T1 and T2, the mean reductions in buccolingual and mesiodistal wound dimensions were 1.30 mm (95% CI: 0.44–2.16; *p* < 0.001) and 1.05 mm (95% CI: 0.20–1.91; *p* = 0.011), respectively.

In the control group, buccolingual and mesiodistal wound dimensions decreased by 4.56 mm (95% CI: 3.71–5.41; *p* < 0.001) and 1.91 mm (95% CI: 1.07–2.75; *p* < 0.001) between T0 and T1, respectively. Between T1 and T2, the mean reductions in buccolingual and mesiodistal wound dimensions were 0.63 mm (95% CI: −0.23–1.50; *p* = 0.229) and 0.66 mm (95% CI: −0.20–1.52; *p* = 0.191), respectively.

The treatment–time interaction was not significant (buccolingual aspect: *p* = 0.389; mesiodistal aspect: *p* = 0.445), indicating that the changes in wound dimensions over time did not significantly differ between the groups.

At T2, two sites (11%) in the control group and three sites (16%) in the test group showed complete wound closure. No significant difference was observed between the groups (*p* = 0.622).

#### 3.3.4. Socket Wound Healing Score (SWHS)

In the test group, the SWHS was reduced by 1.15 (95% CI: 0.74–1.56; *p* < 0.001) and 0.83 (95% CI: 0.41–1.26; *p* < 0.001) between T0 and T1 and between T1 and T2, respectively.

In the control group, the SWHS was reduced by 0.85 (95% CI: 0.44–1.26; *p* < 0.001) and 1.15 (95% CI: 0.73–1.58; *p* < 0.001) between T0 and T1 and between T1 and T2, respectively. 

The treatment–time interaction was not significant (*p* = 0.331), implying that the changes in the SWHS over time did not significantly differ between the groups.

## 4. Discussion

The main objective of this intra-subject RCT was to compare the collagen matrix to the hemostatic gelatin sponge as a socket seal in ARP in terms of soft tissue dimensions. The null hypothesis was rejected since a statistically significant difference in the soft tissue thickness in the center of the site was found between the test and control groups. Sockets sealed with the collagen matrix yielded 0.67 mm thicker soft tissues in the center of the site than sockets sealed with the hemostatic gelatin sponge. The collagen matrix also showed superior results in preserving the buccal soft tissue height when compared to the hemostatic gelatin sponge, with a difference pointing to 0.72 mm. Similarly, there was a trend suggesting the better preservation of the lingual soft tissue height in sockets sealed with a collagen matrix. These findings indicate that a collagen matrix better maintains soft tissue dimensions than a hemostatic gelatin sponge. Although the clinical relevance of these results could not be assessed in this trial, there is high-level evidence associating thicker soft tissues with superior implant esthetics [12] and healthier peri-implant conditions [13].

Changes in the buccal soft tissue profile indicated a reduction in the horizontal soft tissue dimension of approximately 1 mm at 4 months of follow-up. This is in accordance with two recent RCTs using the same methodology [13]. As demonstrated in this study, the collagen matrix failed to influence these soft tissue dynamics, suggesting the need for adjunctive soft tissue augmentation at the moment of implant placement for optimal esthetic outcomes. However, the fact that the collagen matrix resulted in thicker soft tissues in the center of the site may potentially decrease the necessity for adjunctive soft tissue augmentation after implant placement, as the displacement of buccal soft tissues during crown installation could compensate for the loss of buccal soft tissue dimension. The need for soft tissue augmentation after ARP is clinically relevant to study as it is an additional surgical procedure imposing extra morbidity and treatment costs. The potential impact of the socket seal strategy on the necessity for soft tissue augmentation was beyond the scope of this RCT and needs to be assessed in future research. 

Regardless of the socket seal strategy and bone level, significant horizontal bone loss was observed. The most resorption was seen at level −1 mm, and the least was observed at level −5 mm. The collagen matrix did not exert any additional impact on bone preservation. The horizontal bone loss observed is in line with the findings reported in recent studies concerning ARP [2,18,19]. The absence of significant differences in hard tissue outcomes between the test and control groups suggests that both the collagen matrix and hemostatic gelatin sponge effectively sealed the extraction socket and supported bone preservation. 

The reductions in wound dimensions between T0 and T1 were significant in both the test and control groups. However, between T1 and T2, the reduction in the test group remained significant for both buccolingual and mesiodistal wound dimensions, while it was not in the control group. These results suggest a trend favoring better soft tissue healing when a collagen matrix is applied. However, no significant difference was found in the SWHS at any time point between the groups in this study. The wound dimensions and the SWHS in the test group are consistent with earlier findings of Eeckhout et al. [18], investigating ARP using C-DBBM and socket sealing with the same collagen matrix.

To properly understand the findings of the current study, it is essential to acknowledge the following limitations: 

First, the sample size was computed on the primary study outcome. Consequently, the study might have lacked sufficient power to adequately assess differences in secondary outcome variables. 

Second, the primary outcome—the soft tissue thickness in the center of the site—was measured on CBCT. A drawback of CBCT images is the limited soft tissue contrast resolution, primarily caused by the scatter radiation resulting from the imaging of structures outside the field of view [20]. Software that allows for the superimposition of CBCTs of different time points, as well as their alignment with corresponding digital surface models, would provide a more accurate assessment of the soft tissue outline. Indeed, a recent in vitro study by Ferry and co-workers [21] evaluated the accuracy of different measurement methods to assess gingival thickness. Transmucosal probing overestimated soft tissue thickness by 0.22, while DICOM files alone underestimated soft tissue thickness by −0.23. The superimposition of DICOM-STL files showed similar results to the histology assessment with a mean difference of −0.04.

Third, follow-up was limited to 4 months mainly because this study was designed as an intra-subject RCT with basically no limitations with respect to surgical sites. As such, single implant sites with no, one, or two neighboring teeth and adjacent implant sites were included. This renders the current sample too heterogeneous after implant placement for further follow-up. Future studies only including single implant sites, using more accurate soft tissue assessments, longer follow-up, and focusing on the need for adjunctive soft tissue augmentation may provide additional insights into the effects of different socket seal strategies in ARP. Finally, this study compared the effectiveness of two socket seal materials, disregarding the fact that one is about EUR 60 more expensive than the other. Whether the superior effectiveness of a collagen matrix is worth the additional cost, needs to be assessed in a cost-effectiveness analysis. In such studies, it is important to assess the impact of the socket seal material not only on the cost of ARP but also on the total treatment cost.

## 5. Conclusions

The absence of significant differences in hard tissue outcomes suggests that both the collagen matrix and hemostatic gelatin sponge effectively sealed the extraction socket and supported bone preservation. However, the collagen matrix better maintained soft tissue dimensions. The clinical relevance of this finding with respect to the necessity for adjunctive soft tissue augmentation at the moment of implant placement is yet to be studied.

## Figures and Tables

**Table 1:** Secondary outcomes registered on superimposed CBCTs. Estimated marginal means and 95% confidence intervals are shown. bold = statistical significant (*p* < 0.05).

**Width alveolar process at level −1 (mm)**	**T0**	**T3**	
Test group	7.56 (6.68–8.43)	4.89 (4.02–5.77)	*p* = 0.958
Control group	7.77 (6.90–8.65)	5.07 (4.14–6.01)	
**Width alveolar process at level −3 (mm)**			
Test group	8.37 (7.53–9.21)	6.61 (5.76–7.45)	*p* = 0.816
Control group	8.63 (7.79–9.47)	6.75 (5.91–7.60)	
**Width alveolar process at level −5 (mm)**			
Test group	8.78 (7.92–9.64)	7.66 (6.80–8.52)	*p* = 0.679
Control group	8.95 (8.09–9.81)	7.63 (6.78–8.49)	
**Soft tissue height at buccal aspect (mm)**			
Test group	2.39 (1.96–2.82)	2.46 (2.02–2.90)	***p* = 0.034**
Control group	2.63 (2.22–3.04)	1.99 (1.55–2.43)	
**Soft tissue height at lingual aspect (mm)**			
Test group	2.49 (2.04–2.93)	2.80 (2.32–3.29)	*p* = 0.066
Control group	2.58 (2.12–3.03)	2.19 (1.72–2.66)	

## Data Availability

Data are contained within the article.
